# Supplementary figures and images for: Constitutive loss of DNMT3A causes morbid obesity through misregulation of adipogenesis
Source: eLife. 2022 May 30;11:e72359. doi: 10.7554/eLife.72359 (PMC9150890; doi:10.7554/eLife.72359)

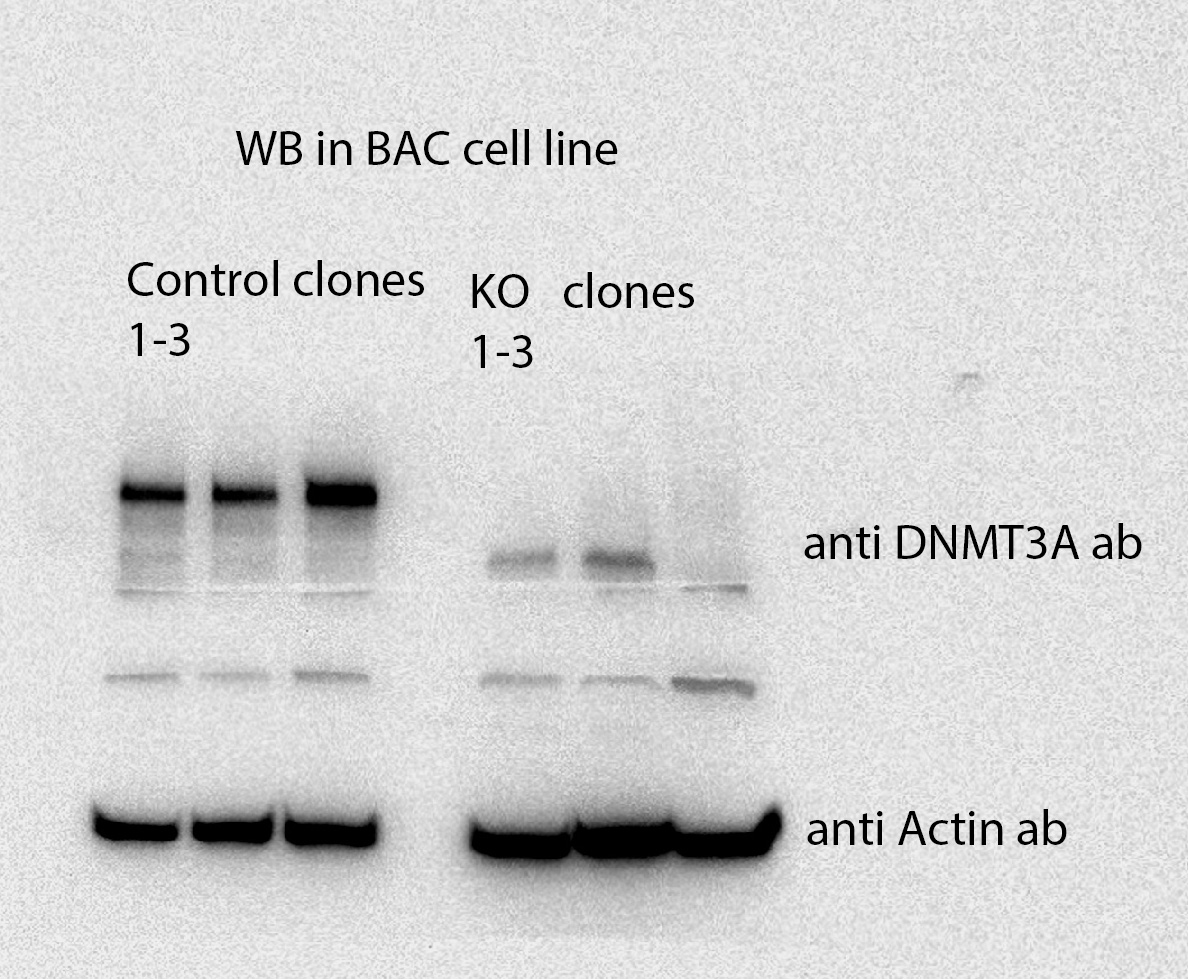

Supplement: Figure 5—source data 3. [file elife-72359-fig5-data3.zip › source_fig5A/Ayala 2017-11-15 11h27m53s annotated.jpg]

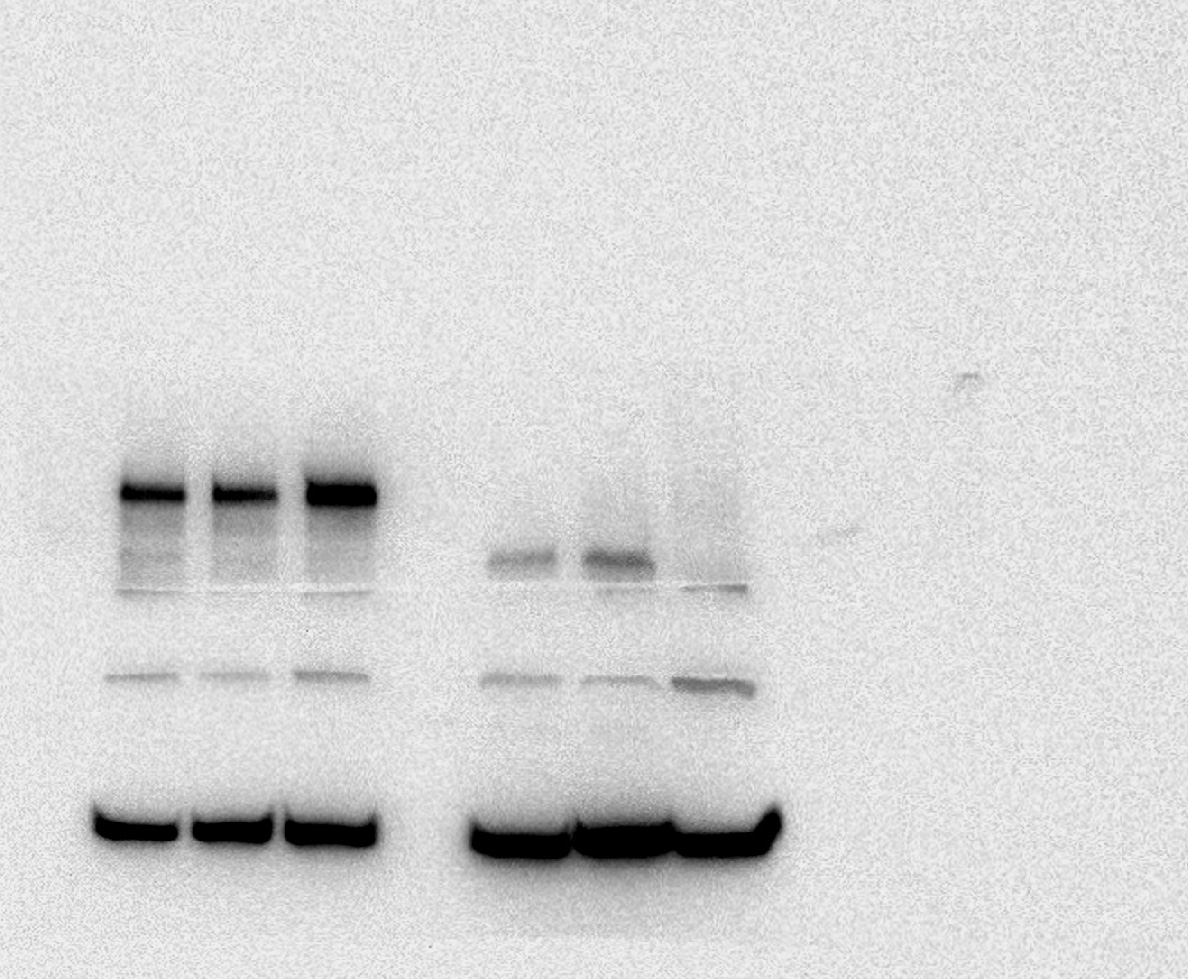

Supplement: Figure 5—source data 3. [file elife-72359-fig5-data3.zip › source_fig5A/Ayala 2017-11-15 11h27m53s.jpg]

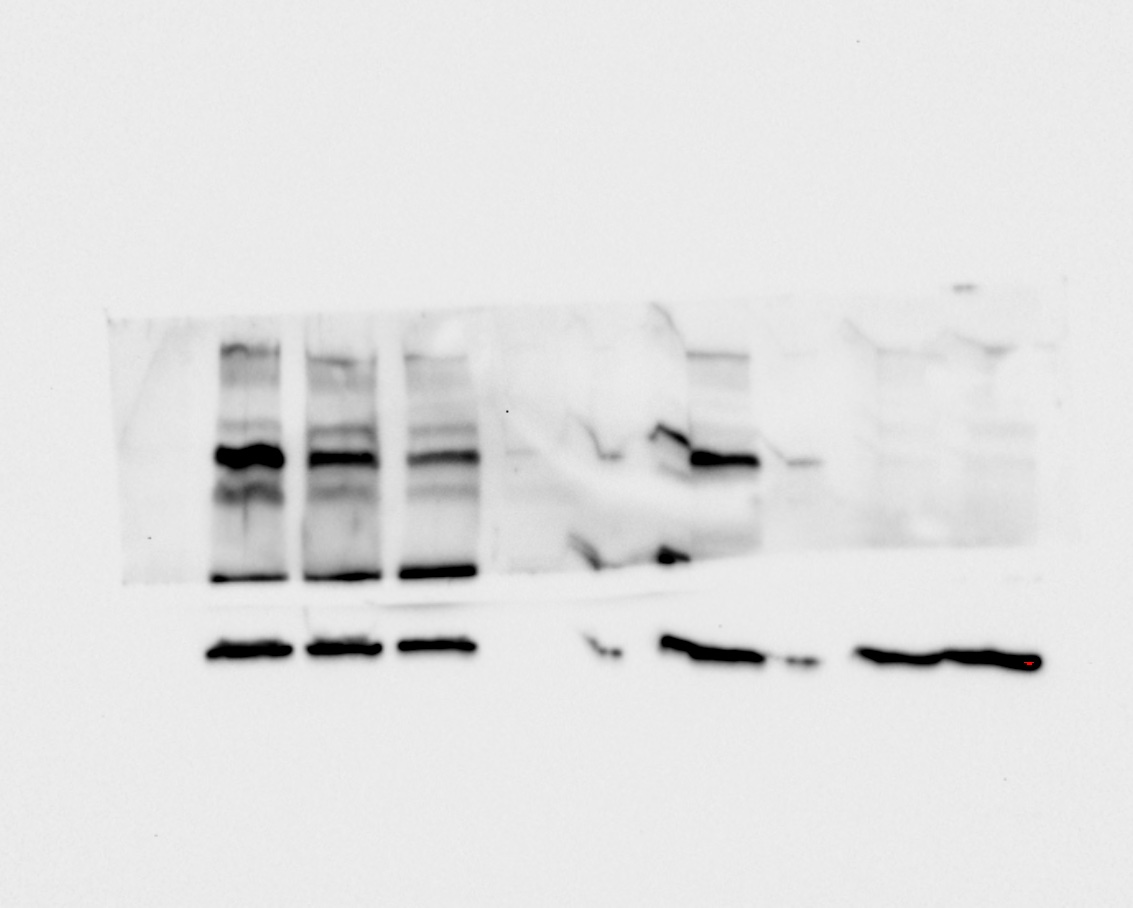

Supplement: Figure 5—figure supplement 1—source data 1. [file elife-72359-fig5-figsupp1-data1.zip › source_supp_fig5A/3T3 .jpg]

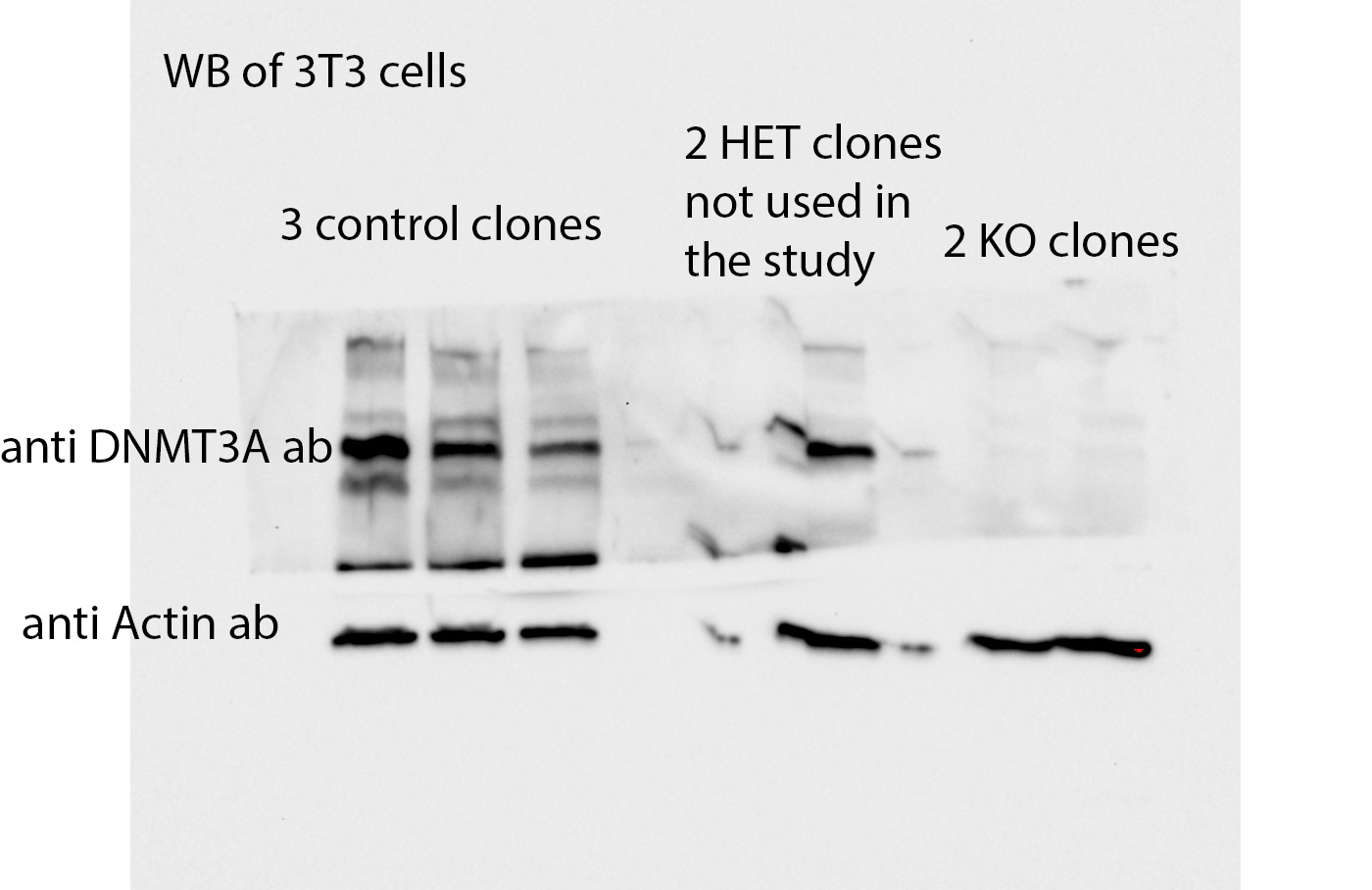

Supplement: Figure 5—figure supplement 1—source data 1. [file elife-72359-fig5-figsupp1-data1.zip › source_supp_fig5A/3T3 annotated.jpg]

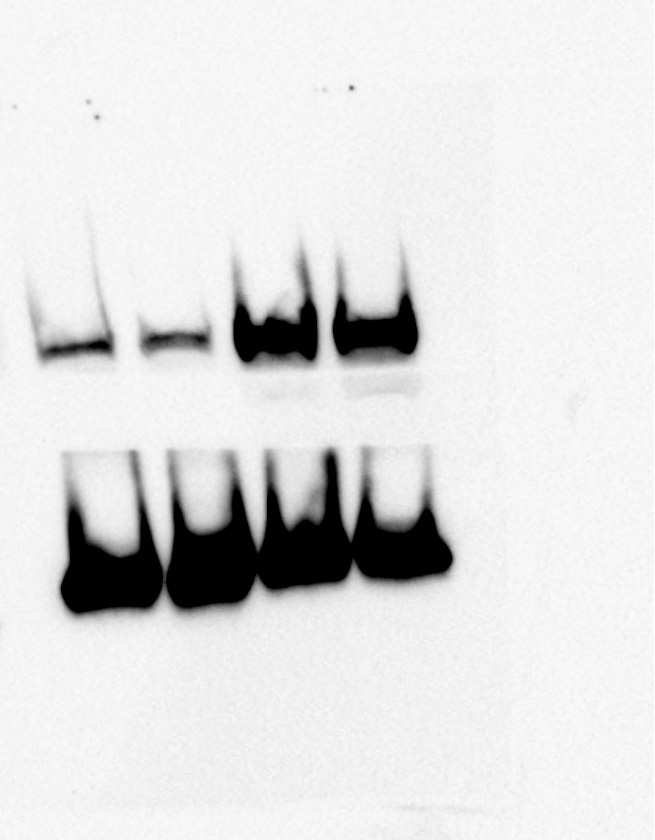

Supplement: Figure 6—figure supplement 1—source data 1. [file elife-72359-fig6-figsupp1-data1.zip › souce_supp_fig6D/HSL_pHSL_WAT.jpg]

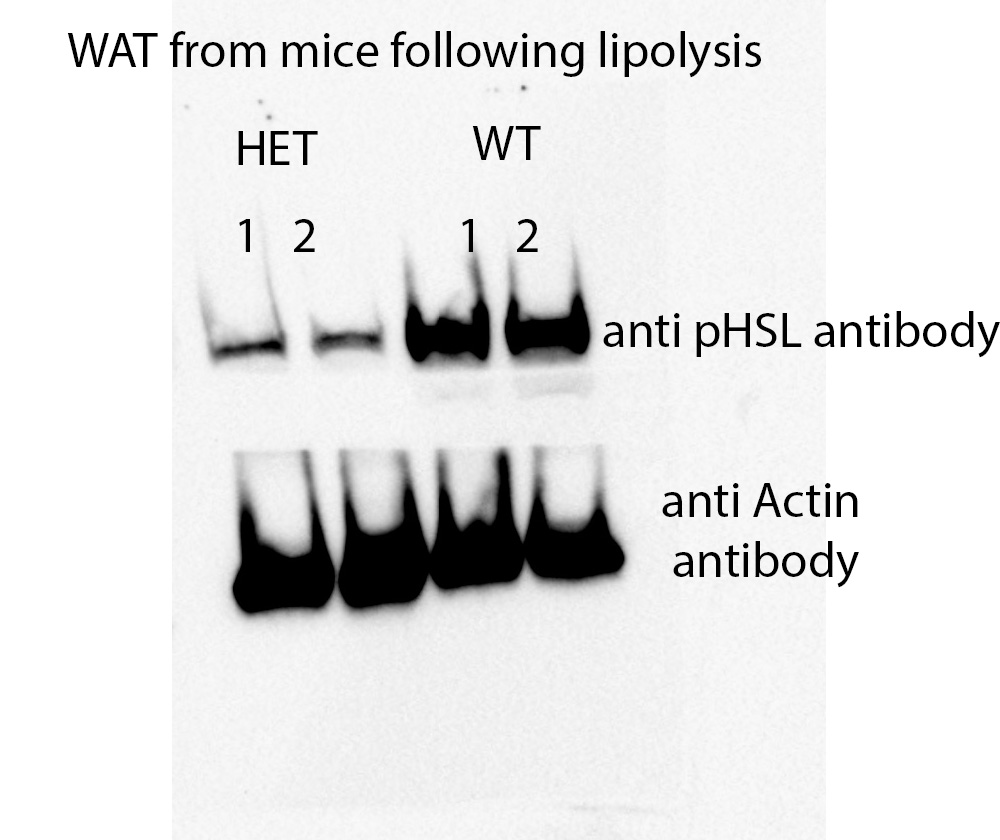

Supplement: Figure 6—figure supplement 1—source data 1. [file elife-72359-fig6-figsupp1-data1.zip › souce_supp_fig6D/HSL_pHSL_WAT annotated.jpg]

WAT from mice following lipolysis

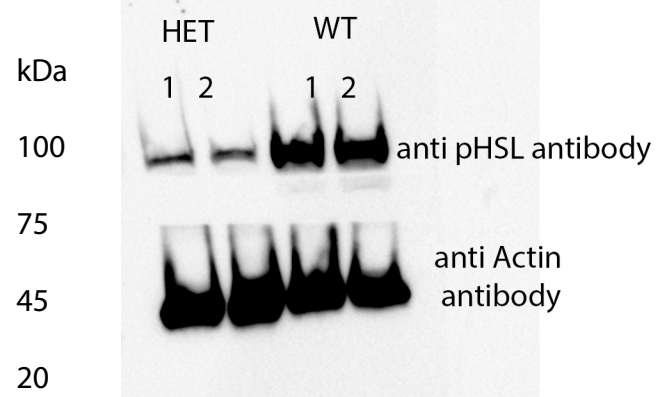

Supplement: Figure 6—figure supplement 1—source data 1. [file elife-72359-fig6-figsupp1-data1.zip › souce_supp_fig6D/HSL_pHSL_WAT annotated.pdf]

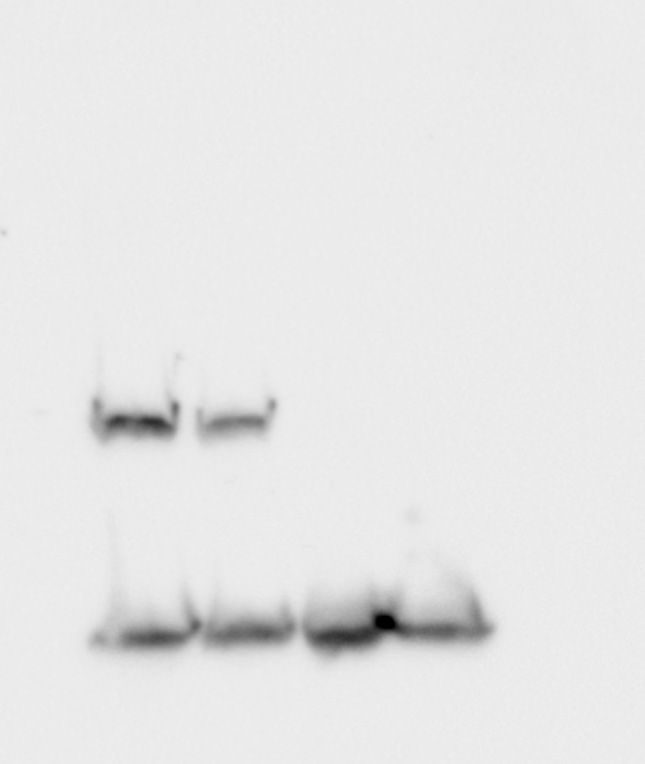

Supplement: Figure 6—figure supplement 1—source data 2. [file elife-72359-fig6-figsupp1-data2.zip › source_supp_fig6E/pHSL_BAT.jpg]

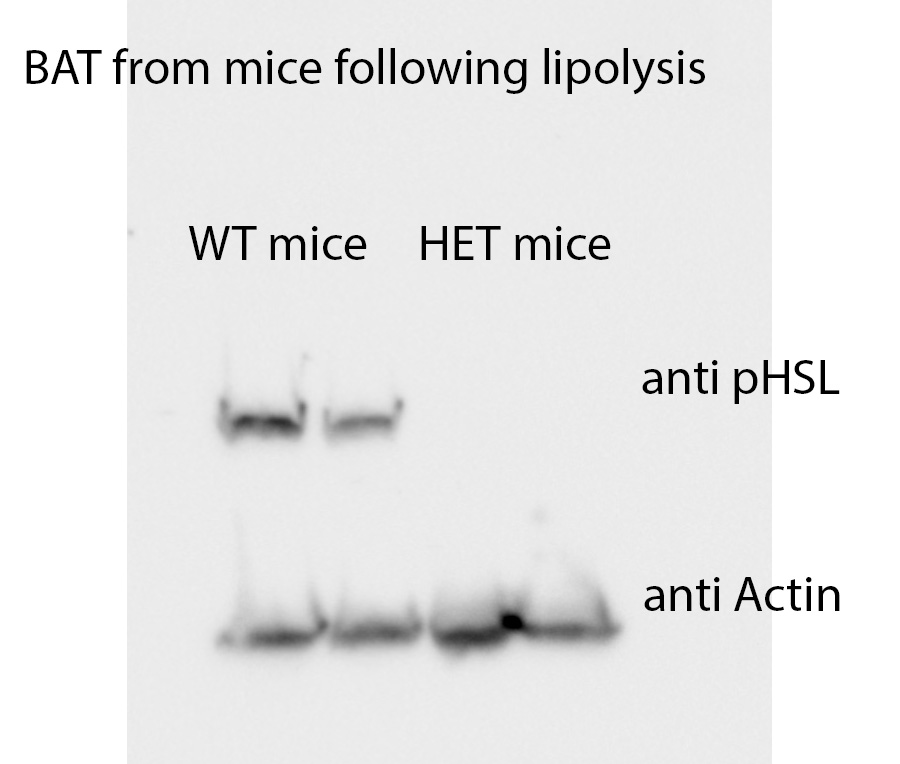

Supplement: Figure 6—figure supplement 1—source data 2. [file elife-72359-fig6-figsupp1-data2.zip › source_supp_fig6E/pHSL_BAT annotated.jpg]
